# From infectious diseases to chronic diseases: the paradigm shift of spatial epidemiology in disease prevention and control

**DOI:** 10.3389/fpubh.2025.1698964

**Published:** 2025-10-14

**Authors:** Ke Hu, Chaojie Li, Xingjin Yang, Shuiping Ou, Xing Zhang, Di Xiao, Mingyang Yu

**Affiliations:** ^1^Xiamen Haicang Hospital, Xiamen, China; ^2^Xingtai Center for Disease Control and Prevention, Xingtai, China; ^3^QianDongNanZhou Center for Disease Control and Prevention, QianDongNanZhou, China; ^4^Honwing Pharma (Guizhou) Company Limited, QianDongNanZhou, China; ^5^Nanjing Lishui Dongping Street Health Center, Nanjing, China; ^6^Community Health Service Center of Jiuxian Tongliang District, Chongqing, China; ^7^Fuwai Central China Cardiovascular Hospital, Zhengzhou, China

**Keywords:** spatial epidemiology, infectious disease prevention and control, chronic disease management, spatial analysis methods, data aggregation scale, geographic information system

## Abstract

Spatial epidemiology, as an important branch of epidemiology, has undergone a significant paradigm shift from infectious disease prevention and control to chronic disease management. This paper systematically reviews the application progress of spatial epidemiology in the study of infectious diseases (e.g., malaria, HIV) and chronic diseases (e.g., cancer, cardiovascular diseases), focusing on its role in identifying spatial distribution patterns of diseases, assessing environmental exposures, and supporting health decision-making. The paper compares the differences in data characteristics, analytical methods, and modeling strategies between infectious and chronic diseases, and discusses the impact of multi-scale analysis, data aggregation, and the Modifiable Areal Unit Problem on research results. Furthermore, this paper reviews the innovative value of Geographic Information Systems, remote sensing technology, mobile positioning, and multi-source data fusion in promoting precision public health practices. Finally, the article points out the current challenges faced by spatial epidemiology in privacy ethics, causal inference, and model robustness, and prospects future directions such as AI-enabled multi-omics integration and spatial decision support systems under global health governance.

## 1 Introduction

### 1.1 Definition and development of spatial epidemiology

Spatial epidemiology, as an important sub-discipline of epidemiology, has undergone significant development and evolution over the past 25 years (1). This discipline primarily describes and analyzes the spatial distribution patterns of disease risk factors, incidence, and mortality, investigating their associations with demographic characteristics, socioeconomic status, environmental exposures, health behaviors, and genetic risk factors (2). Its origins can be traced back to a critical period of development in spatial statistics and geographical technologies, which provided the methodological foundation for spatial disease analysis (1). With the evolution of technologies such as Geographic Information Systems (GIS), spatial epidemiology has expanded from its initial focus on infectious diseases to the field of chronic diseases, forming a complete theoretical system and technical framework (3).

### 1.2 Paradigm shift from infectious disease control to chronic disease management

The global shift in disease spectrum has driven a major transformation in the research paradigm of spatial epidemiology. Traditionally, this field was primarily applied to the prevention and control of infectious diseases such as malaria and Human Immunodeficiency Virus (HIV) (4). However, with the increasing burden of chronic diseases, spatial analysis methods have been successfully extended to the study of non-communicable diseases (4). This shift reflects the transition of epidemiology from infectious to chronic diseases (5), while also revealing the complex associations between infectious pathogens and the occurrence of chronic diseases, such as the link between enteroviruses and various chronic diseases in children (6). It is noteworthy that the current public health field faces a complex situation where infectious and chronic degenerative diseases coexist and are interrelated (7), presenting new research challenges and opportunities for spatial epidemiology.

### 1.3 Value of methodological innovation through interdisciplinary integration

The innovative development of spatial epidemiology highly depends on multidisciplinary integration. This field integrates spatial information technologies such as GIS, remote sensing technology, and mobile positioning data (8–11), while also incorporating the life-course epidemiology framework to study the long-term effects of biological, environmental, behavioral, and psychosocial factors on chronic disease risk (12). Methodologically, spatial analysis techniques can not only identify spatial clustering patterns of diseases (13) but also assess environmental exposure risks (14) and optimize the allocation of prevention and control resources (15). This multidisciplinary integration promotes the development of precision public health practices (16), providing a spatial dimension of scientific evidence for decision-making in the prevention and control of both infectious and chronic diseases (16, 17). However, academia still needs to strengthen mechanisms for translating innovative technologies into practical prevention and control capabilities to bridge the gap between theoretical research and implementation (18).

## 2 Application progress of spatial epidemiology in infectious disease prevention and control

### 2.1 Establishment and development of spatial transmission models for infectious diseases

The development of spatial transmission models for infectious diseases has evolved from simple statistical descriptions to complex spatiotemporal predictions. In recent years, with advances in GIS and remote sensing technology, spatial modeling techniques have been able to integrate multi-source data to simulate the transmission dynamics of pathogens (8). Modern spatial transmission models not only include traditional point pattern analysis and areal data analysis but also incorporate innovative methods such as space–time scan statistics, significantly improving the prediction accuracy of disease diffusion paths (19, 20). These models show unique advantages in evaluating the effects of different prevention and control measures, providing scientific basis for public health decision-making. It is noteworthy that the main challenge in current model development lies in how to translate the results of fine spatiotemporal scale analyses into actionable recommendations for practical prevention and control actions (18).

### 2.2 Case studies of spatial analysis for classic infectious diseases such as malaria/HIV

Spatial analysis provides a critical perspective for understanding the distribution patterns and intrinsic relationships of classic infectious diseases such as malaria and HIV. In a study in Zimbabwe, spatial scan statistics revealed no spatial overlap in the primary clusters of HIV/AIDS and malaria, but spatial overlap of secondary clusters was found in five districts in the northern and eastern parts of the country. This finding provides a basis for targeted prevention and control; for example, screening and intervention measures for both diseases can be integrated in the overlapping areas (21). A study in Ethiopia found spatial co-distribution of HIV, tuberculosis, and malaria prevalence in some areas, which was significantly associated with healthcare accessibility, demographic, and climatic factors (22). It is noteworthy that the application of spatial analysis is not limited to classical infectious diseases. Taking cutaneous leishmaniasis in Iran as an example, analysis confirms that disease hotspots are significantly associated with environmental factors such as high altitude (600–1800 meters) and specific land surface temperatures (23).

Specific cases demonstrate that spatial analysis methods can effectively identify disease transmission patterns. For example, when using Kulldorff’s spatial scan statistic to identify high HIV/AIDS prevalence areas in Zimbabwe, data obtained from the District Health Information System (DHIS) revealed that HIV was more widely distributed than malaria (21). In malaria research, spatial autocorrelation analysis using a discrete Poisson model revealed significant spatiotemporal clustering in specific geographical areas (*p* < 0.05) (24). These cases confirm that spatial analysis can support the development of precise prevention and control strategies. For instance, Chiapas State in Mexico used geospatial tools to identify associations between malaria risk and socio-environmental factors (25), while China combined molecular epidemiology with spatial analysis to track the mobility pathways of HIV-infected individuals (26).

### 2.3 Spatial epidemiological analysis and early warning systems in public health emergencies

In public health emergencies, spatial epidemiological early warning systems integrate GIS, spatial statistics, and multi-source real-time data to achieve dynamic monitoring of spatiotemporal disease transmission patterns, risk prediction, and targeted intervention. The core of these systems lies in transforming abstract case data into intuitive spatial risk maps, with their functions mainly manifested in three aspects:

First, in terms of early warning and source tracing, the system can utilize methods like spatial scan statistics for rapid anomaly detection. For example, during a Salmonella outbreak in New York City, the system successfully located the source restaurant before the official announcement, demonstrating its prominent value for early warning (27).

Second, regarding transmission pattern analysis and risk assessment, relevant studies on the COVID-19 pandemic in Wuhan through spatiotemporal analysis revealed the correlation between spatial clustering characteristics of cases and urban infrastructure such as transportation and population density, providing crucial data support for geography-informed emergency decision-making (28).

Finally, for dynamic risk assessment and optimization of control strategies, the system needs the capability to identify changes in transmission patterns. For instance, research in the Kenyan highlands showed a lack of consistency in malaria incidence hotspots over a 10-year period, suggesting that static hotspot maps have limitations in areas with low and unstable transmission, necessitating the establishment of dynamic, continuously re-evaluated surveillance mechanisms (29).

Therefore, spatial epidemiological early warning systems deeply integrate the “location” dimension into decision-making, marking a strategic shift in public health management from passive response to active prediction.

## 3 Expanded application of spatial epidemiology in chronic disease research

### 3.1 Geographic distribution patterns of cancer and environmental exposure assessment

As a major health threat among chronic diseases today, the application of spatial epidemiology in cancer has shifted from descriptive analysis to assessing the practical impact on prevention strategies. Research indicates that geographic variation in cancer outcomes and their risk factors is significantly associated with area socioeconomic status and community composition (30). Environmental exposure assessment is a core component of spatial cancer analysis, including studies on the spatial association between the geographic distribution of environmental carcinogens (such as air pollution, industrial emissions) and cancer incidence rates. For example, in the Campania region of Italy, spatial autocorrelation techniques confirmed the spatial clustering of cancer mortality and environmental health hazards (such as industrial pollution), directly contributing to the delineation of priority intervention areas in the locality (31).

Environmental exposure assessment uses multi-scale methods (such as JoinPoint, SaTScan, and spatial regression models) to reveal spatial associations between air pollution (PM) and cancer incidence (32, 33). Research confirms a high degree of spatial overlap between PM exposure and lung cancer incidence. For instance, a study in Guangzhou, China, found higher PM exposure risks in densely populated areas, where lung cancer incidence rates were also significantly higher (34). Another study in Beijing identified a potential spatial association between PM and cancer incidence, suggesting the incorporation of PM exposure assessment into environmental epidemiological monitoring (33). The US SEER registry system, as a high-quality population-based data infrastructure, facilitates sophisticated spatial analyses of cancer patterns. For example, one study using SEER data employed spatial statistical methods to identify geographic disparities and service coverage gaps in prostate cancer care, highlighting how such registry systems can pinpoint areas with potential environmental and healthcare access challenges (35).

The translation of spatial epidemiology into policy confronts several challenges. First, although advanced methods like Bayesian spatial analysis can leverage cancer registry data for tailored interventions (36), technical limitations persist. Second, geocoding errors, for instance, can lead to exposure misclassification, requiring standardized protocols to improve data reliability (37, 38). To address these limitations and better capture the complexities of dynamic human mobility, the field is increasingly adopting Geographic Artificial Intelligence (GeoAI). This approach integrates high-resolution environmental data with mobile monitoring to refine exposure assessment (39, 40).

### 3.2 Spatial clustering analysis of cardiovascular diseases

Spatial epidemiological research on Cardiovascular Diseases (CVDs) has evolved from theoretical description to supporting practical interventions. Using GIS and spatial statistical techniques (such as Moran’s I, hotspot analysis, and geographically weighted regression), studies have not only identified high-risk clusters in southern South Africa (41, 42) but also revealed spatial associations between population density and congenital heart disease hotspots (43). These findings directly guide targeted interventions: for example, in Ulsan, South Korea, spatial clustering analysis of social determinants provided a basis for developing regional policies addressing cardiovascular health disparities (44); while in Colombia’s Pacific region, spatiotemporal mortality analysis linked municipal socioeconomic indicators to CVD burden, optimizing resource allocation strategies (45). Recent cases show that spatial hotspot analysis can precisely identify areas requiring enhanced comprehensive interventions (46), such as US-based research on integrated prevention for CVD and cancer based on county-level mortality clusters (47), and the identification of high-priority intervention areas in Tanzania through retrospective spatiotemporal models (48). These practices confirm that spatial epidemiological methods can effectively promote the transition of public health strategies from “universal coverage” to “precision prevention and control” (49, 50).

### 3.3 Spatial heterogeneity of social determinants of chronic diseases

Differences in the spatial distribution of chronic diseases often reflect geographical inequalities in social determinants. Research shows a significant negative correlation between the area deprivation index (SDI) and chronic disease risk, with residents in areas with better socioeconomic conditions showing lower overall chronic disease risk (51). Spatial analysis methods can effectively capture the non-uniform distribution characteristics of these social factors and their complex associations with health outcomes. Particularly noteworthy is that data from the China Health and Retirement Longitudinal Study (CHARLS) reveal that multimorbidity among the older adults population in China does not follow a random distribution but demonstrates identifiable spatial clustering patterns, such as high prevalence of specific combinations like hypertension with arthritis or gastric diseases (52). By integrating GIS with socioeconomic data, a more comprehensive understanding of the spatial heterogeneity of the chronic disease burden can be achieved, providing decision support for precision public health interventions (49, 53). This multi-dimensional spatial analysis framework is of great value for developing targeted chronic disease prevention and control strategies (54).

## 4 Methodological comparison of spatial analysis for different disease types

### 4.1 Differences in spatial data characteristics between infectious and chronic diseases

As shown in Table 1, infectious and chronic diseases exhibit significant differences in spatial data characteristics. Spatial data for infectious diseases typically manifest as point distribution patterns, with clear spatiotemporal transmission paths and clustering features (55). Such data often contain precise time and location information of onset, capable of reflecting the dynamic process of pathogen transmission through human contact or vectors (56, 57). In contrast, spatial data for chronic diseases more often present areal distribution characteristics, related to long-term cumulative effects of environmental exposures, socioeconomic factors, etc. (58, 59). Chronic disease data usually lack precise time stamps, and their spatial distribution more reflects regional differences in risk factors (60).

**Table 1:** Comparison of spatial data characteristics between infectious and chronic diseases.

Feature	Infectious diseases	Chronic diseases	Applicable scenarios and selection rationale
Spatial Distribution Pattern	Point distribution with clear spatiotemporal transmission paths	Areal distribution reflecting regional risk factors	Infectious diseases require point pattern analysis for dynamic transmission tracking; chronic diseases suit areal analysis for long-term exposure assessment.
Temporal dynamics	Highly dynamic, requiring real-time/near-real-time monitoring	Relatively stable, suitable for cross-sectional or long-term trend analysis	Infectious disease models need short time windows; chronic disease studies use extended periods to identify stable clusters.
Data sources	Mandatory reporting systems	Disease registries, health surveys	Infectious disease data are actively reported; chronic disease data are often passively collected.
Key analytical methods	Kernel density estimation, Ripley’s K, spatial scan statistics	Patial Autocorrelation, GWR, small area estimation	Point pattern methods fit infectious diseases; areal methods account for socioeconomic and environmental exposures in chronic diseases.

Spatial data for infectious diseases are highly dynamic and require support from real-time or near-real-time monitoring systems (61). Conversely, spatial data for chronic diseases exhibit relatively stable geographical distribution patterns, making them more suitable for cross-sectional or long-term trend analysis (62). In terms of data sources, infectious disease data mostly come from mandatory reporting systems, while chronic disease data rely more on passive collection methods such as disease registries and health surveys (63).

### 4.2 Technical choices and limitations of point pattern analysis and areal data analysis

Point pattern analysis methods for infectious disease research primarily include Kernel Density Estimation (64) (used to visualize hotspot areas of case clustering), Ripley’s K function (65) (identifies specific spatial scales of case clustering), and Spatial Scan Statistics (66) (detects statistically significant disease clusters using a moving window). These methods can effectively identify case cluster areas and transmission sources, and are suitable for individual-level data with precise geocoding. In studies of infectious diseases like malaria and HIV, point pattern analysis has been successfully applied to identify high-risk areas and evaluate intervention effectiveness (21).

However, these methods also have limitations. For example, the results of Kernel Density Estimation are highly dependent on bandwidth selection (67), which is subjective. Although spatial scan statistics can provide statistical significance, the shape and size of its scanning window need to be predefined (68), which may not perfectly match the true shape of disease clusters, thus affecting detection accuracy.

Areal data analysis is more suitable for chronic disease research. Commonly used methods include Spatial Autocorrelation Analysis [e.g., Global/Local Moran’s I, used to quantify disease spatial clustering across the entire region or in local areas (69)], Geographically Weighted Regression [GWR, used to explore how the effect strength of risk factors varies across geographical space (70)], and Small Area Estimation [uses Bayesian models to smooth data and address the instability of rates in small sample areas (71)]. This approach divides the study area into several spatial units (e.g., administrative divisions) to analyze the spatial variation of disease rates or risk factors. In cardiovascular disease and cancer research, areal analysis helps reveal the spatial heterogeneity of environmental exposures and socioeconomic factors (72–75).

The main limitation of areal analysis is the Modifiable Areal Unit Problem (MAUP) (76), meaning the results can be significantly influenced by the way regional units are divided and their scale (e.g., county, city, province). Additionally, ecological fallacy isa potential risk (77), where correlations derived from aggregated data may not accurately infer individual-level relationships.

Technical selection must consider data availability and research objectives. Point pattern analysis requires precise geographic coordinates but may face privacy protection restrictions (78); areal analysis can utilize routinely collected aggregated data but may be affected by MAUP (79) (Table 2).

**Table 2:** Technical comparison of point pattern analysis vs. areal data analysis.

Aspect	Point pattern analysis	Areal data analysis	Applicable scenarios and selection rationale
Data requirement	Precise geographic coordinates	Aggregated data by spatial units (e.g., administrative divisions)	Use point pattern when individual-level geocoded data are available; use areal analysis when only aggregated data are accessible.
Typical methods	Kernel density, spatial scan statistics	Global/Local Moran’s I, GWR, Bayesian smoothing	Point pattern detects clusters and sources; areal analysis reveals spatial heterogeneity and ecological associations.
Limitations	Bandwidth subjectivity, predefined window shape	Modifiable Areal Unit Problem (MAUP), ecological fallacy	Point pattern may over-smooth; areal analysis is sensitive to zoning and scale effects.
Application examples	Malaria/HIV hotspot detection	Cancer mortality mapping, CVD risk area identification	Infectious diseases benefit from point-based clustering; chronic diseases use areal units for policy-relevant mapping.

### 4.3 Parameter optimization strategies and challenges for spatiotemporal scan statistics

Spatiotemporal scan statistics have important applications both in early warning for infectious diseases and long-term monitoring of chronic diseases. Parameter optimization must consider disease type characteristics: for infectious diseases, shorter time windows (e.g., days to weeks) should be set to capture rapid transmission processes (80); for chronic diseases, the observation period needs to be extended (e.g., months to years) to identify stable spatial clustering patterns (81).

The choice of spatial scanning window shape also requires differentiation: infectious disease studies often use circular windows to detect local outbreaks (82); chronic disease studies can use elliptical or irregularly shaped windows to match the geographical distribution of environmental exposures (83). The maximum scanning window size should be set to detect meaningful clusters while ensuring statistical power, but not so large as to obscure the internal real structure or produce difficult-to-interpret results.

Multiple comparison correction strategies also need adjustment: early warning systems for infectious diseases might use a less strict significance level (e.g., *p* < 0.1) to increase sensitivity; chronic disease studies should employ strict correction (e.g., *p* < 0.01) to reduce the false positive rate (84).

It is noteworthy that a key challenge for spatiotemporal scan statistics lies in their computational complexity and the methods for correcting multiple comparisons, which can impose a computational burden (85, 86). Furthermore, the method typically assumes the population at risk is uniform within the scanning window (82). This assumption may not hold in real-world scenarios with uneven population density or large-scale population movement, potentially leading to detection bias. Recently developed Bayesian spatiotemporal modeling methods (e.g., Bayesian spatiotemporal scan statistics) (87, 88), which can better handle small area data instability and spatial correlation by incorporating prior distributions, show advantages in chronic disease studies and offer new directions for addressing these limitations.

## 5 Impact mechanism of data aggregation scale on research results

### 5.1 Empirical research on the MAUP

MAUP, which is widespread in spatial data analysis, refers to the sensitivity of analytical results to the arbitrarily chosen spatial aggregation units during data measurement (76). This issue is particularly prominent in disease mapping. When high-resolution spatial health data are aggregated for reasons such as privacy protection, the resulting “single-aggregation disease map” relies entirely on the selected aggregation units to represent the underlying data (89). The MAUP manifests specifically in two effects: the zoning effect, which occurs when the boundaries of analytical units are altered, and the scale effect, which arises when the level of aggregation is changed (90, 91). Empirical studies have shown that in the analysis of cancer mortality rates in Portugal from 2009 to 2013, choices regarding the level of aggregation can lead to significantly different research outcomes (92). Furthermore, data from COVID-19 wastewater monitoring projects in New York State have confirmed the substantial impact of the MAUP scale effect on epidemiological surveillance results (93).

### 5.2 Key role of multi-scale analysis in exposure assessment

To address the challenges posed by MAUP, multi-scale analysis methods have become an important strategy in exposure assessment (91). This approach establishes linkage models at different spatial scales, connecting convolution models at different scale levels using shared random effects (94). Research conducted in low-population-density areas of Australia showed that exploring intermediate aggregation levels and multi-scale methods can better capture subtle disease dynamics (91). Multi-scale models allow the integration of variables acting at different scales into a single model while minimizing information loss. This method is not only suitable for specific ecological contexts but also has application value in broader spatial analysis. Particularly in environmental epidemiology studies, multi-scale analysis helps address the issue of unmeasured confounders arising from the use of administratively aggregated data (95).

### 5.3 Integration methods for individual-level and group-level data

Spatial epidemiological data are often arranged hierarchically, where individuals are classified into smaller units, which are in turn grouped into larger units. This structure can produce contextual effects (96). To address this issue, researchers have proposed shared multilevel models that can simultaneously handle the scale effects generated by aggregating data from smaller units into larger units (96). In data downscaling methods, the *a priori* chosen scale and shape significantly influence the results, requiring researchers to carefully consider integration strategies for individual-level and group-level data (97). In traffic injury research, converting sparse collision point data into a continuous risk surface via Kernel Density Estimation (KDE), combined with Multiscale Geographically Weighted Regression (MGWR) methods, effectively overcame the impact of MAUP on point data aggregation (98). Furthermore, Exploratory Spatial Data Analysis (ESDA) methods provide a flexible solution to scale issues at different jurisdictional levels by iteratively assessing changes in spatial patterns during the process of upgrading high-resolution maps (99).

## 6 Technological innovation and multi-source data fusion

### 6.1 Technological evolution of GIS

GIS, as a core technical tool for spatial epidemiology, have undergone significant technological innovation over the past 25 years. Their development trajectory has kept pace with technological advances in the fields of spatial statistics and geography (1). As presented in Table 3, modern GIS technology can integrate core functions such as geocoding, distance calculation, and spatial interpolation, providing powerful spatial analysis capabilities for epidemiological research (3). In the field of cancer research, GIScience has significantly improved the accuracy of environmental exposure assessment by integrating multiple geographical perspectives and spatial analysis methods (100). It is noteworthy that the spatial analysis functions of GIS, combined with genetic projection pursuit models, can effectively improve the reliability of assessment results (101). With the popularization of visualization tools, the role of GIS in public health decision support systems is becoming increasingly prominent (49).

**Table 3:** Evolution and applications of core technologies in spatial epidemiology.

Technology	Key functions	Applications in spatial epidemiology	Applicable scenarios and selection rationale
Geographic information systems (GIS)	Geocoding, distance calculation, spatial interpolation	Disease mapping, resource allocation, environmental exposure assessment	GIS integrates spatial data and supports visual decision-making; essential for both infectious and chronic disease studies.
Remote sensing (RS) and mobile positioning	Environmental monitoring, human mobility tracking	Air pollution exposure, infectious disease spread modeling	RS provides large-scale environmental data; mobile data capture real-time population movements for dynamic modeling.
Spatial big data and AI	Multi-source data fusion, real-time prediction	Precision public health, outbreak early warning	Useful when high-volume, high-variety data are available; AI enhances predictive accuracy and intervention targeting.

### 6.2 Application breakthroughs of remote sensing and mobile positioning data

The combination of Remote Sensing (RS) technology and mobile positioning data has created a new paradigm for disease surveillance. In an empirical study in Hunan Province, change patch data from remote sensing images showed a strong correlation with Real-Time Kinematic (RTK) positioning data (102). This technological integration provides effective indicators for geographic information updates (102). In air pollution research, seasonal spatiotemporal modeling methods based on remote sensing data and GIS achieved analysis of PM distribution characteristics at a 1 km grid level (103). New digital data sources such as mobile phone call detail records and geotagged tweets are reshaping infectious disease surveillance systems (104). Particularly noteworthy is the cascaded parallel Long Short-Term Memory-Conditional Random Field (LSTM-CRF) model proposed in landslide prediction research, which demonstrates the innovative value of multi-source data fusion by integrating remote sensing images and GIS’s big spatial data processing capabilities (105).

### 6.3 Spatial big data-driven precision public health practice

Spatial big data, characterized by its Volume, Velocity, and Variety, is driving public health practice toward precision (106). New data sources such as medical claims data, mobile phone signaling, and social media geotags provide real-time dynamic information for infectious disease surveillance that traditional systems cannot capture (104). In an empirical study in Shenzhen, researchers combined remote sensing data with geographic big data to construct a neighborhood-scale urban vitality assessment model using the random forest method (107). The introduction of artificial intelligence technology further enhances the analytical capabilities of spatial big data. Large-scale geographic health datasets based on electronic health records provide new ways to trace patients’ geographical exposure history (108). These technological advances enable epidemiological research to break through traditional data limitations and play a greater role in health risk communication and cross-scale public health coordination (104).

## 7 Discussion

### 7.1 Current challenges and controversies

#### 7.1.1 Ethical balance between privacy protection and data sharing

The core ethical dilemma in spatial epidemiological research lies in balancing the protection of individual privacy with the need for scientific data sharing. On one hand, regulations such as HIPAA (Health Insurance Portability and Accountability Act) have ambiguous provisions regarding the protection of geographic data, which restricts the sharing and use of spatial health data (109). On the other hand, traditional methods (e.g., geographic masking), while capable of protecting individual location privacy, may compromise critical spatial statistical features (78, 110). Current solutions include: (1) Federated learning (FL), which addresses data silos through distributed model training (sharing only parameters rather than raw data), but requires integration with techniques such as differential privacy (DP) to enhance security (111–113); (2) Privacy-preserving geostatistical models (e.g., Zip4 aggregation), which achieve anonymization while maintaining spatial analytical accuracy (114, 115). However, these methods still necessitate a trade-off between privacy strength and data utility and require interdisciplinary collaboration to optimize implementation frameworks.

#### 7.1.2 Limitations of causal inference from spatial analysis results

Spatial epidemiology has significant methodological limitations in causal inference. Ecological fallacy is one of the most prominent issues, occurring when researchers erroneously extrapolate conclusions derived from spatial aggregate analysis at the group level to the individual level (116). In health exposure modeling, particularly disease mapping studies, ecological fallacy manifests as a systematic deviation between the relationship of aggregated disease incidence and average exposure level at the areal unit level, and the relationship between individual disease events and relevant individual exposure levels (77). Recent research shows that about 67% of multivariate model studies have causal inference defects, and only 16% of studies select variables based on a causal inference framework (117). Although the counterfactual causal inference framework provides new ideas for answering ecological causal questions (118), spatial epidemiology has not fully adapted to the contemporary emphasis in epidemiology on causal inference and intervention research (1). This requires researchers to handle spatial correlation analysis results more cautiously (119) and develop synthetic population data tools that can integrate multi-level causal structures (120).

#### 7.1.3 Risks of model overfitting and ecological fallacy

Spatial epidemiological models face dual risks of overfitting and ecological fallacy. Unreasonable prior assumptions can seriously affect multiple key aspects of epidemiological research, including inter-regional transmission rates, importance of transmission paths, number of transmission events, and pathogen ancestry relationships (121). In spatial modeling of diseases such as schistosomiasis, ecological fallacy can lead to unreliability in identifying at-risk populations (116). Furthermore, the widespread phenomenon of the “Table 2 Fallacy” (i.e., misinterpretation of multivariate model results) in multivariate model studies occurs in up to 67% of orthopedic literature (117), highlighting the issue of standardization in the application of statistical methods in spatial analysis. To mitigate these risks, researchers need to adopt more robust prior distributions to enhance topic relevance analysis, and can employ modeling approaches based on known at-risk populations at the regional level and independent environmental monitoring data to avoid reliance on individual-level exposure information or random allocation assumptions (77). At the same time, it should be recognized that individual-level data is irreplaceable for assessing causality affecting individuals (120), which provides important implications for the design of spatial epidemiological studies.

### 7.2 Future development directions

#### 7.2.1 AI-enabled real-time spatial early warning systems

The World Health Organization’s “Global Initiative on AI for Health” (GI-AI4H) is coordinating the development of governance standards for AI in health, with particular focus on implementation in low- and middle-income countries (122). GeoAI, as an emerging interdisciplinary field, integrates spatial science, machine learning, and big data computing technologies, enabling the extraction of key knowledge from spatial big data (39). In the field of infectious disease surveillance, applications of spatial AI combining real-time data from IoT devices with GIS are building multi-dimensional disease surveillance decision support systems (123, 124). Future research should focus on developing spatiotemporal prediction models based on deep learning algorithms, integrating multi-source data such as environment, climate, and population, to achieve proactive outbreak management and precise intervention (124).

#### 7.2.2 Integration pathways for multi-omics data and spatial analysis

Emerging technologies such as spatial transcriptomics and spatial proteomics are providing unprecedented spatial resolution for studying tumor heterogeneity (125, 126). AI-driven multimodal models can decipher the complex molecular interactions underlying cell behavior and tissue dynamics. Deep learning algorithms, in particular, show great potential in biomedical image analysis tasks such as cell segmentation, phenotype recognition, and cancer prognosis prediction (125). In tumor microenvironment research, AI-based spatial transcriptome analysis helps understand the mechanisms of cell–cell interactions (127). Future development directions include establishing multi-center collaborative networks, promoting the integrated application of spatial omics technologies and computational tools, and facilitating the translation from research on tumor spatial heterogeneity to precision treatment plans (128, 129).

#### 7.2.3 Building spatial decision support systems in global health governance

Global AI governance should prioritize the principle of equity, particularly by empowering Global South nations to lead the development of solutions (130). In terms of ethical governance, a balanced mechanism that integrates both privacy protection and data sharing ought to be established, utilizing tools such as Participatory Geographic Information Systems (PGIS) to incorporate interdisciplinary data (124, 131). GeoAI technologies provide new multi-scale analytical tools for health disparity research, enabling improved interpretation of the spatial heterogeneity in health determinants at both individual and regional levels (9, 132). Future efforts should focus on building a global health decision-support system that incorporates environmental exposure assessment, social determinants analysis, and real-time monitoring data, while also addressing algorithmic biases stemming from Western-centric cognitive frameworks (39, 130).

## 8 Conclusion

Spatial epidemiology has undergone a significant paradigm shift, evolving from infectious disease prevention and control to chronic disease management. This evolution highlights its strong adaptability in addressing complex public health challenges.

The application of technologies such as Geographic Information Systems and remote sensing has greatly enhanced our understanding of disease spatial patterns. However, the field still faces challenges including privacy ethics and causal inference.

Looking ahead, the integration of artificial intelligence (AI) with spatial epidemiology promises revolutionary advances. AI can not only build real-time early warning systems by integrating multi-source data to improve response capabilities for infectious diseases but also promote the development of precision public health through the analysis of multi-omics data. Furthermore, these technologies hold potential for application in often-neglected animal disease research, which is crucial for implementing the “One Health” concept and comprehensively safeguarding public health.

In the new era of data-driven approaches, spatial epidemiology will provide critical support for building a more resilient global public health system through technological innovation.

## References

[1] Morrison CN Mair CF Bates L Duncan DT Branas CC Bushover BR. Defining spatial epidemiology: a systematic review and Re-orientation. Epidemiology. (2024) 35:542–55. doi: 10.1097/ede.0000000000001738, PMID: 38534176 PMC11196201

[2] Tang Z Sun Q Pan J Xie M Wang Z Lin X. Air pollution's numerical, spatial, and temporal heterogeneous impacts on childhood hand, foot and mouth disease: a multi-model county-level study from China. BMC Public Health. (2024) 24:2825. doi: 10.1186/s12889-024-20342-x, PMID: 39407189 PMC11479553

[3] Kirby RS Delmelle E Eberth JM. Advances in spatial epidemiology and geographic information systems. Ann Epidemiol. (2017) 27:1–9. doi: 10.1016/j.annepidem.2016.12.001, PMID: 28081893

[4] Cuadros DF Li J Musuka G Awad SF. Spatial epidemiology of diabetes: methods and insights. World J Diabetes. (2021) 12:1042–56. doi: 10.4239/wjd.v12.i7.1042, PMID: 34326953 PMC8311478

[5] Celentano DD Platz E Mehta SH. The centennial of the Department of Epidemiology at Johns Hopkins Bloomberg School of Public Health: a century of epidemiologic discovery and education. Am J Epidemiol. (2019) 188:2043–8. doi: 10.1093/aje/kwz176, PMID: 31509178 PMC7306280

[6] Tseng JJ Lin CH Lin MC. Long-term outcomes of Pediatric enterovirus infection in Taiwan: a population-based cohort study. Front Pediatr. (2020) 8:285. doi: 10.3389/fped.2020.00285, PMID: 32596191 PMC7303813

[7] Saldana-Jimenez F Almaguer-Martinez FJ Hernandez-Cabrera F Morales-Vidales JA Soto-Rocha MVI Walle-Garcia O. Impact and evolution of risk factors associated with hospitalization and mortality due to COVID-19 during the six epidemic waves in Mexico. Heliyon. (2024) 10:e27962. doi: 10.1016/j.heliyon.2024.e27962, PMID: 38510039 PMC10950712

[8] Qiu J Li X Zhu H Xiao F. Spatial epidemiology and its role in prevention and control of swine viral disease. Animals. (2024) 14:14. doi: 10.3390/ani14192814, PMID: 39409763 PMC11476123

[9] Zhang Z Manjourides J Cohen T Hu Y Jiang Q. Spatial measurement errors in the field of spatial epidemiology. Int J Health Geogr. (2016) 15:21. doi: 10.1186/s12942-016-0049-5, PMID: 27368370 PMC4930612

[10] Mehmood K Bao Y Mushtaq S Saifullah Khan MA Siddique N. Perspectives from remote sensing to investigate the COVID-19 pandemic: a future-oriented approach. Front Public Health. (2022) 10:938811. doi: 10.3389/fpubh.2022.938811 35958871 PMC9360797

[11] Jia P Xue H Yin L Stein A Wang M Wang Y. Spatial Technologies in Obesity Research: current applications and future promise. Trends Endocrinol Metab. (2019) 30:211–23. doi: 10.1016/j.tem.2018.12.003, PMID: 30712979

[12] Jia P Dong W Yang S Zhan Z Tu L Lai S. Spatial Lifecourse epidemiology and infectious disease research. Trends Parasitol. (2020) 36:235–8. doi: 10.1016/j.pt.2019.12.012, PMID: 32044243 PMC7172117

[13] Diaz D Vazquez-Polanco AM Argueta-Donohue J Stephens CR Jimenez-Trejo F Ceballos-Liceaga SE. Incidence of intestinal infectious diseases due to Protozoa and Bacteria in Mexico: analysis of National Surveillance Records from 2003 to 2012. Biomed Res Int. (2018) 2018:1–12. doi: 10.1155/2018/2893012, PMID: 30112374 PMC6077666

[14] Kim D. Exploratory study on the spatial relationship between emerging infectious diseases and urban characteristics: cases from Korea. Sustain Cities Soc. (2021) 66:102672. doi: 10.1016/j.scs.2020.102672, PMID: 33520608 PMC7828747

[15] Li C Chen K Yang K Li J Zhong Y Yu H. Progress on application of spatial epidemiology in ophthalmology. Front Public Health. (2022) 10:936715. doi: 10.3389/fpubh.2022.936715, PMID: 36033806 PMC9399620

[16] Lan T Cheng M Lin YD Jiang LY Chen N Zhu MT. Self-reported critical gaps in the essential knowledge and capacity of spatial epidemiology between the current university education and competency-oriented professional demands in preparing for a future pandemic among public health postgraduates in China: a nationwide cross-sectional survey. BMC Med Educ. (2023) 23:646. doi: 10.1186/s12909-023-04578-6, PMID: 37679696 PMC10485961

[17] Byun HG Lee N Hwang SS. A systematic review of spatial and Spatio-temporal analyses in public Health Research in Korea. J Prev Med Public Health. (2021) 54:301–8. doi: 10.3961/jpmph.21.160, PMID: 34649392 PMC8517372

[18] Tatem AJ. Innovation to impact in spatial epidemiology. BMC Med. (2018) 16:209. doi: 10.1186/s12916-018-1205-5, PMID: 30424754 PMC6234695

[19] Huang J Yan W Li H Hu S Hao Z Li L. Development of two-dimension epidemic prediction model. Infect Dis Model. (2025) 10:1190–207. doi: 10.1016/j.idm.2025.06.009, PMID: 40689266 PMC12271440

[20] Hundessa SH Williams G Li S Guo J Chen L Zhang W. Spatial and space-time distribution of plasmodium vivax and plasmodium falciparum malaria in China, 2005-2014. Malar J. (2016) 15:595. doi: 10.1186/s12936-016-1646-2, PMID: 27993171 PMC5168843

[21] Gwitira I Murwira A Mberikunashe J Masocha M. Spatial overlaps in the distribution of HIV/AIDS and malaria in Zimbabwe. BMC Infect Dis. (2018) 18:598. doi: 10.1186/s12879-018-3513-y, PMID: 30482166 PMC6260695

[22] Alene KA Elagali A Barth DD Rumisha SF Amratia P Weiss DJ. Spatial codistribution of HIV, tuberculosis and malaria in Ethiopia. BMJ Glob Health. (2022) 7:e007599. doi: 10.1136/bmjgh-2021-007599, PMID: 35217531 PMC8867247

[23] Ramezankhani R Sajjadi N Nezakati Esmaeilzadeh R Jozi SA Shirzadi MR. Spatial analysis of cutaneous leishmaniasis in an endemic area of Iran based on environmental factors. Geospat Health. (2017) 12:578. doi: 10.4081/gh.2017.578, PMID: 29239561

[24] Gwitira I Mukonoweshuro M Mapako G Shekede MD Chirenda J Mberikunashe J. Spatial and spatio-temporal analysis of malaria cases in Zimbabwe. Infect Dis Poverty. (2020) 9:146. doi: 10.1186/s40249-020-00764-6, PMID: 33092651 PMC7584089

[25] Castillo-Salgado C. Geo-epidemiologic mapping in the new public health surveillance. The malaria case in Chiapas, Mexico, 2002. Gac Med Mex. (2017) 153:S5–S12. doi: 10.24875/gmm.M000001 29099113

[26] Yuan D Liu S Ouyang F Ai W Shi L Liu X. Prevention and control are not a regional matter: a spatial correlation and molecular linkage analysis based on newly reported HIV/AIDS patients in 2021 in Jiangsu, China. Viruses. (2023) 15:2053. doi: 10.3390/v15102053, PMID: 37896830 PMC10612072

[27] Latash J Greene SK Stavinsky F Li S McConnell JA Novak J. Salmonellosis outbreak detected by automated spatiotemporal analysis - new York City, May-June 2019. MMWR Morb Mortal Wkly Rep. (2020) 69:815–9. doi: 10.15585/mmwr.mm6926a2, PMID: 32614808 PMC7332097

[28] Liu W Wang D Hua S Xie C Wang B Qiu W. Spatiotemporal analysis of COVID-19 outbreaks in Wuhan, China. Sci Rep. (2021) 11:13648. doi: 10.1038/s41598-021-93020-2, PMID: 34211038 PMC8249501

[29] Hamre KES Hodges JS Ayodo G John CC. Lack of consistent malaria incidence hotspots in a Highland Kenyan area during a 10-year period of very low and unstable transmission. Am J Trop Med Hyg. (2020) 103:2198–207. doi: 10.4269/ajtmh.19-0821, PMID: 33124534 PMC7695093

[30] Torres AZ Phelan-Emrick D Castillo-Salgado C. Evaluating Neighborhood correlates and geospatial distribution of breast, cervical, and colorectal Cancer incidence. Front Oncol. (2018) 8:471. doi: 10.3389/fonc.2018.00471, PMID: 30425965 PMC6218580

[31] Agovino M Aprile MC Garofalo A Mariani A. Cancer mortality rates and spillover effects among different areas: a case study in Campania (southern Italy). Soc Sci Med. (2018) 204:67–83. doi: 10.1016/j.socscimed.2018.03.027, PMID: 29587157

[32] Gao Y Zhang M Liu G Lin H Huang H Xie H. Historical data analysis and future prediction of lung cancer in Zhejiang province, China. Sci Rep. (2025) 15:21813. doi: 10.1038/s41598-025-07200-5, PMID: 40594686 PMC12218021

[33] Ouyang W Gao B Cheng H Hao Z Wu N. Exposure inequality assessment for PM(2.5) and the potential association with environmental health in Beijing. Sci Total Environ. (2018) 635:769–78. doi: 10.1016/j.scitotenv.2018.04.190, PMID: 29710600

[34] Fan W Xu L Zheng H. Using multisource data to assess PM(2.5) exposure and spatial analysis of lung Cancer in Guangzhou, China. Int J Environ Res Public Health. (2022) 19:19. doi: 10.3390/ijerph19052629, PMID: 35270346 PMC8910196

[35] Modi PK Ward KC Filson CP. Characteristics of prostate cancer patients captured by facility-based versus geography-based cancer registries. Urol Oncol. (2023) 41:324.e1–7. doi: 10.1016/j.urolonc.2023.04.011, PMID: 37150737

[36] Saint-Jacques N Brown PE Purcell J Rainham DG Terashima M Dummer TJB. The Nova Scotia community Cancer matrix: a geospatial tool to support cancer prevention. Soc Sci Med. (2023) 330:116038. doi: 10.1016/j.socscimed.2023.116038, PMID: 37390806

[37] Kinnee EJ Tripathy S Schinasi L Shmool JLC Sheffield PE Holguin F. Geocoding error, spatial uncertainty, and implications for exposure assessment and environmental epidemiology. Int J Environ Res Public Health. (2020) 17:17. doi: 10.3390/ijerph17165845, PMID: 32806682 PMC7459468

[38] Fisher JA Spaur M Buller ID Flory AR Beane Freeman LE Hofmann JN. Spatial heterogeneity in positional errors: a comparison of two residential geocoding efforts in the agricultural health study. Int J Environ Res Public Health. (2021) 18:18. doi: 10.3390/ijerph18041637, PMID: 33572119 PMC7915413

[39] VoPham T Hart JE Laden F Chiang YY. Emerging trends in geospatial artificial intelligence (geoAI): potential applications for environmental epidemiology. Environ Health. (2018) 17:40. doi: 10.1186/s12940-018-0386-x, PMID: 29665858 PMC5905121

[40] Dedele A Miskinyte A Grazuleviciene R. The impact of particulate matter on allergy risk among adults: integrated exposure assessment. Environ Sci Pollut Res Int. (2019) 26:10070–82. doi: 10.1007/s11356-019-04442-5, PMID: 30756350

[41] Darikwa TB Manda SO. Spatial co-clustering of cardiovascular diseases and select risk factors among adults in South Africa. Int J Environ Res Public Health. (2020) 17:17. doi: 10.3390/ijerph17103583, PMID: 32443772 PMC7277617

[42] Iyanda A Ade-Oni A Omiyefa S. A geographic perspective of the association between physical activity and cardiovascular health: a need for community-level intervention. J Prev Interv Community. (2024):1–30. doi: 10.1080/10852352.2024.2415162, PMID: 39422301

[43] Klein JH Cuneo B Howley L Kavanaugh-McHugh A Taylor C Chaves AH. Geospatial distribution of prenatally and postnatally diagnosed congenital heart disease: implications for equitable care from a Fetal heart society research collaborative study. J Pediatr. (2024) 273:114120. doi: 10.1016/j.jpeds.2024.114120, PMID: 38815740

[44] Lee SH Ock M Moon S Song EK. The impact of clusters with distinct social determinants by a two-step cluster analysis on cardiovascular health. J Cardiovasc Nurs. (2025). doi: 10.1097/jcn.0000000000001238, PMID: 40631938

[45] Perez-Florez M Achcar JA. Socioeconomic inequalities in mortality due to cardiovascular diseases: Pacific region of Colombia, 2002-2015. Ciênc Saúde Colet. (2021) 26:5201–14. doi: 10.1590/1413-812320212611.3.02562020, PMID: 34787211

[46] Oinam B Anand V Kajal RK. A spatiotemporal geographic information system-based assessment of human immunodeficiency virus/acquired immune deficiency syndrome distribution in Manipur, India. Indian J Public Health. (2021) 65:362–8. doi: 10.4103/ijph.IJPH_1308_20, PMID: 34975079

[47] Amin RW Steinmetz J. Spatial clusters of life expectancy and association with cardiovascular disease mortality and cancer mortality in the contiguous United States: 1980-2014. Geospat Health. (2019) 14:14. doi: 10.4081/gh.2019.733, PMID: 31099524

[48] Sianga BE Mbago MC Msengwa AS. The distribution of cardiovascular diseases in Tanzania: a spatio-temporal investigation. Geospat Health. (2024) 19:19. doi: 10.4081/gh.2024.1307, PMID: 39259195

[49] Eberth JM Kramer MR Delmelle EM Kirby RS. What is the place for space in epidemiology? Ann Epidemiol. (2021) 64:41–6. doi: 10.1016/j.annepidem.2021.08.022, PMID: 34530128

[50] Arauzo-Carod JM. A first insight about spatial dimension of COVID-19: analysis at municipality level. J Public Health (Oxf). (2021) 43:98–106. doi: 10.1093/pubmed/fdaa140, PMID: 32808010 PMC7454828

[51] Seconda L Baudry J Alles B Touvier M Hercberg S Pointereau P. Prospective associations between sustainable dietary pattern assessed with the sustainable diet index (SDI) and risk of cancer and cardiovascular diseases in the French NutriNet-Sante cohort. Eur J Epidemiol. (2020) 35:471–81. doi: 10.1007/s10654-020-00619-2, PMID: 32140936

[52] Guo X Zhao B Chen T Hao B Yang T Xu H. Multimorbidity in the elderly in China based on the China health and retirement longitudinal study. PLoS One. (2021) 16:e0255908. doi: 10.1371/journal.pone.0255908, PMID: 34352011 PMC8341534

[53] Liu L Nagar G Diarra O Shosanya S Sharma G Afesumeh D. Epidemiology for public health practice: the application of spatial epidemiology. World J Diabetes. (2022) 13:584–6. doi: 10.4239/wjd.v13.i7.584, PMID: 36051429 PMC9329838

[54] Parbs JR Srinivasan S Pustz J Bayly R Shrestha S Lewis O. The optimization of harm reduction services in Massachusetts through the use of GIS: location-allocation analyses, 2019-2021. Prev Med. (2024) 186:108088. doi: 10.1016/j.ypmed.2024.108088, PMID: 39084414

[55] Lawson AB. Bayesian disease mapping: Hierarchical Modeling in spatial epidemiology. 3rd ed. New York: Chapman and Hall/CRC (2018). 486 p.

[56] Stoddard ST Morrison AC Vazquez-Prokopec GM Paz Soldan V Kochel TJ Kitron U. The role of human movement in the transmission of vector-borne pathogens. PLoS Negl Trop Dis. (2009) 3:e481. doi: 10.1371/journal.pntd.0000481, PMID: 19621090 PMC2710008

[57] Kulldorff M. A spatial scan statistic. Commun Stat. (1997) 26:1481–96. doi: 10.1080/03610929708831995

[58] Briggs DJ. A framework for integrated environmental health impact assessment of systemic risks. Environ Health. (2008) 7:61. doi: 10.1186/1476-069x-7-61, PMID: 19038020 PMC2621147

[59] Pickett KE Pearl M. Multilevel analyses of neighbourhood socioeconomic context and health outcomes: a critical review. J Epidemiol Community Health. (2001) 55:111–22. doi: 10.1136/jech.55.2.111, PMID: 11154250 PMC1731829

[60] Danaei G Singh GM Paciorek CJ Lin JK Cowan MJ Finucane MM. The global cardiovascular risk transition: associations of four metabolic risk factors with national income, urbanization, and western diet in 1980 and 2008. Circulation. (2013) 127:1502e1491-1498:1493–502. doi: 10.1161/circulationaha.113.001470 PMC4181853 23481623

[61] Brownstein JS Freifeld CC Madoff LC. Digital disease detection--harnessing the Web for public health surveillance. N Engl J Med. (2009) 360:2153–7. doi: 10.1056/NEJMp0900702 19423867 PMC2917042

[62] Elliott P Wartenberg D. Spatial epidemiology: current approaches and future challenges. Environ Health Perspect. (2004) 112:998–1006. doi: 10.1289/ehp.6735, PMID: 15198920 PMC1247193

[63] Lee LM Thacker SB. The cornerstone of public health practice: public health surveillance. MMWR Suppl. (2011) 60:15–21. 21976162

[64] Liu L Zhu HH Huang JL Han XP Qian MB Yang K. Spatiotemporal epidemiological characteristics of human clonorchiasis in China. Acta Trop. (2025) 271:107832. doi: 10.1016/j.actatropica.2025.107832, PMID: 40935004

[65] Self S Overby A Zgodic A White D McLain A Dyckman C. A hypothesis test for detecting distance-specific clustering and dispersion in areal data. Spat Stat. (2023) 55:100575. doi: 10.1016/j.spasta.2023.100757, PMID: 37396190 PMC10312012

[66] Li X-Z Wang J-F Yang W-Z Li Z-J Lai S-J. A spatial scan statistic for nonisotropic two-level risk cluster. Stat Med. (2012) 31:177–87. doi: 10.1002/sim.4341, PMID: 21850654

[67] Ruckthongsook W Tiwari C Oppong JR Natesan P. Evaluation of threshold selection methods for adaptive kernel density estimation in disease mapping. Int J Health Geogr. (2018) 17:10. doi: 10.1186/s12942-018-0129-9, PMID: 29739415 PMC5938815

[68] Wang L Li X Zhang Z Yuan H Lu P Li Y. Comparing circular and flexibly-shaped scan statistics for disease clustering detection. Front Public Health. (2024) 12:1432645. doi: 10.3389/fpubh.2024.1432645, PMID: 39845679 PMC11750662

[69] Hu K Li C Yang X Xiao D Zhang X Yu M. Spatial stratified heterogeneity of mumps incidence in China: a Geodetector-based analysis of driving factors. Front Public Health. (2025) 13:1637288. doi: 10.3389/fpubh.2025.1637288, PMID: 40873982 PMC12378461

[70] Hu K Zhang X Yang X Yu M. A study on the spatial distribution of life expectancy and its air pollution factors in China based on geographically weighted regression. Front Public Health. (2025) 13:1565744. doi: 10.3389/fpubh.2025.1565744, PMID: 40356832 PMC12068413

[71] Gause EL Schumacher AE Ellyson AM Withers SD Mayer JD Rowhani-Rahbar A. An introduction to bayesian spatial smoothing methods for disease mapping: modeling county firearm suicide mortality rates. Am J Epidemiol. (2024) 193:1002–9. doi: 10.1093/aje/kwae005, PMID: 38375682 PMC12096304

[72] Deng S Liang J Peng Y Liu W Su J Zhu S. Spatial analysis of the impact of urban built environment on cardiovascular diseases: a case study in Xixiangtang, China. BMC Public Health. (2024) 24:2368. doi: 10.1186/s12889-024-19884-x, PMID: 39217314 PMC11366168

[73] Baptista EA Queiroz BL. Spatial analysis of cardiovascular mortality and associated factors around the world. BMC Public Health. (2022) 22:1556. doi: 10.1186/s12889-022-13955-7, PMID: 35974391 PMC9380346

[74] Bian Y Yu X Zhang J Zhao Y Zheng M Tang C. Geographical analysis of malignant tumor incidence and treatment in China. Sci Rep. (2025) 15:32049. doi: 10.1038/s41598-025-17452-w, PMID: 40890268 PMC12402056

[75] Bhakkan-Mambir B Deloumeaux J Luce D. Geographical variations of cancer incidence in Guadeloupe, French West Indies. BMC Cancer. (2022) 22:783. doi: 10.1186/s12885-022-09886-6, PMID: 35843938 PMC9290250

[76] Tuson M Yap M Kok MR Murray K Turlach B Whyatt D. Incorporating geography into a new generalized theoretical and statistical framework addressing the modifiable areal unit problem. Int J Health Geogr. (2019) 18:6. doi: 10.1186/s12942-019-0170-3, PMID: 30917821 PMC6437958

[77] Wang F Wang J Gelfand A Li F. Accommodating the ecological fallacy in disease mapping in the absence of individual exposures. Stat Med. (2017) 36:4930–42. doi: 10.1002/sim.7494, PMID: 28929501

[78] Broen K Trangucci R Zelner J. Measuring the impact of spatial perturbations on the relationship between data privacy and validity of descriptive statistics. Int J Health Geogr. (2021) 20:3. doi: 10.1186/s12942-020-00256-8, PMID: 33413390 PMC7788553

[79] Jahan F Haque S Hogg J Price A Hassan C Areed W. Assessing the influence of the modifiable areal unit problem on Bayesian disease mapping in Queensland, Australia. PLoS One. (2025) 20:e0313079. doi: 10.1371/journal.pone.0313079, PMID: 39874284 PMC11774366

[80] Kazazian L Lima Neto AS Sousa GS Nascimento OJD Castro MC. Spatiotemporal transmission dynamics of co-circulating dengue, zika, and chikungunya viruses in Fortaleza, Brazil: 2011-2017. PLoS Negl Trop Dis. (2020) 14:e0008760. doi: 10.1371/journal.pntd.0008760, PMID: 33104708 PMC7644107

[81] Xu L Deng Y. Spatiotemporal pattern evolution and driving factors of brucellosis in China, 2003-2019. Int J Environ Res Public Health. (2022) 19:19. doi: 10.3390/ijerph191610082, PMID: 36011728 PMC9408399

[82] Tango T. Spatial scan statistics can be dangerous. Stat Methods Med Res. (2021) 30:75–86. doi: 10.1177/0962280220930562, PMID: 33595399

[83] Roche LM Niu X Stroup AM Henry KA. Disparities in female breast Cancer stage at diagnosis in New Jersey: a spatial-temporal analysis. J Public Health Manag Pract. (2017) 23:477–86. doi: 10.1097/phh.0000000000000524, PMID: 28430705 PMC5548504

[84] Zhang M Dai X Chen G Liu Y Wu Z Ding C. The association between spatial-temporal distribution of prostate Cancer and environmental factors in mainland China. Cancer Epidemiol Biomarkers Prev. (2023) 32:208–16. doi: 10.1158/1055-9965.Epi-22-0799, PMID: 36484983

[85] Han J Zhu L Kulldorff M Hostovich S Stinchcomb DG Tatalovich Z. Using Gini coefficient to determining optimal cluster reporting sizes for spatial scan statistics. Int J Health Geogr. (2016) 15:27. doi: 10.1186/s12942-016-0056-6, PMID: 27488416 PMC4971627

[86] Takahashi K Shimadzu H. Multiple-cluster detection test for purely temporal disease clustering: integration of scan statistics and generalized linear models. PLoS One. (2018) 13:e0207821. doi: 10.1371/journal.pone.0207821, PMID: 30462741 PMC6249023

[87] Self S Nolan M. A Bayesian spatial scan statistic for multinomial data. Stat Probab Lett. (2024) 206:206. doi: 10.1016/j.spl.2023.110005, PMID: 38283114 PMC10817011

[88] Ayubi E Niksiar S Keshtpour Amlashi Z Talebi-Ghane E. Spatiotemporal mapping of colorectal and gastric Cancer incidence in Hamadan Province, Western Iran (2010-2019). J Res Health Sci. (2025) 25:e00650. doi: 10.34172/jrhs.2025.185, PMID: 40259653 PMC12009487

[89] Tuson M Yap M Kok MR Boruff B Murray K Vickery A. Overcoming inefficiencies arising due to the impact of the modifiable areal unit problem on single-aggregation disease maps. Int J Health Geogr. (2020) 19:40. doi: 10.1186/s12942-020-00236-y, PMID: 33010800 PMC7532343

[90] Briz-Redón Á. A bayesian shared-effects modeling framework to quantify the modifiable areal unit problem. Spat Stat. (2022) 51:100689. doi: 10.1016/j.spasta.2022.100689

[91] Haque S Price A Mengersen K Hu W. Evaluating the impact of the modifiable areal unit problem on ecological model inference: a case study of COVID-19 data in Queensland, Australia. Infect Dis Model. (2025) 10:1002–19. doi: 10.1016/j.idm.2025.05.003, PMID: 40495898 PMC12148736

[92] Roquette R Nunes B Painho M. The relevance of spatial aggregation level and of applied methods in the analysis of geographical distribution of cancer mortality in mainland Portugal (2009-2013). Popul Health Metrics. (2018) 16:6. doi: 10.1186/s12963-018-0164-6, PMID: 29587794 PMC5870502

[93] Zhu Y Hill DT Zhou Y Larsen DA. The effect of the modifiable areal unit problem (MAUP) on spatial aggregation of COVID-19 wastewater surveillance data. Sci Total Environ. (2024) 957:177676. doi: 10.1016/j.scitotenv.2024.177676, PMID: 39571813

[94] Aregay M Lawson AB Faes C Kirby RS Carroll R Watjou K. Multiscale measurement error models for aggregated small area health data. Stat Methods Med Res. (2016) 25:1201–23. doi: 10.1177/0962280216661094, PMID: 27566773 PMC5437596

[95] Li G Denise H Diggle P Grimsley J Holmes C James D. A spatio-temporal framework for modelling wastewater concentration during the COVID-19 pandemic. Environ Int. (2023) 172:107765. doi: 10.1016/j.envint.2023.107765, PMID: 36709674 PMC9847331

[96] Aregay M Lawson AB Faes C Kirby RS Carroll R Watjou K. Comparing multilevel and multiscale convolution models for small area aggregated health data. Spatial Spatio-temporal Epidemiology. (2017) 22:39–49. doi: 10.1016/j.sste.2017.06.001, PMID: 28760266

[97] Da Re D Gilbert M Chaiban C Bourguignon P Thanapongtharm W Robinson TP. Downscaling livestock census data using multivariate predictive models: sensitivity to modifiable areal unit problem. PLoS One. (2020) 15:e0221070. doi: 10.1371/journal.pone.0221070, PMID: 31986146 PMC6984718

[98] Kuo PF Sulistyah UD Putra IGB Lord D. Exploring the spatial relationship of e-bike and motorcycle crashes: implications for risk reduction. J Saf Res. (2024) 88:199–216. doi: 10.1016/j.jsr.2023.11.007, PMID: 38485363

[99] Zen M Candiago S Schirpke U Egarter Vigl L Giupponi C. Upscaling ecosystem service maps to administrative levels: beyond scale mismatches. Sci Total Environ. (2019) 660:1565–75. doi: 10.1016/j.scitotenv.2019.01.087, PMID: 30743948

[100] Sahar L Foster SL Sherman RL Henry KA Goldberg DW Stinchcomb DG. GIScience and cancer: state of the art and trends for cancer surveillance and epidemiology. Cancer. (2019) 125:2544–60. doi: 10.1002/cncr.32052, PMID: 31145834 PMC6625915

[101] Jiang H Fan G Zhang D Zhang S Fan Y. Evaluation of eco-environmental quality for the coal-mining region using multi-source data. Sci Rep. (2022) 12:6623. doi: 10.1038/s41598-022-09795-5, PMID: 35459255 PMC9033819

[102] Zeng X Zhou F Ao M Chen C. Research on geographic information update index of RTK location big data. Sci Rep. (2025) 15:4494. doi: 10.1038/s41598-025-88252-5, PMID: 39915502 PMC11802873

[103] Xu D Lin W Gao J Jiang Y Li L Gao F. PM(2.5) exposure and health risk assessment using remote sensing data and GIS. Int J Environ Res Public Health. (2022) 19:19. doi: 10.3390/ijerph19106154, PMID: 35627689 PMC9141174

[104] Lee EC Asher JM Goldlust S Kraemer JD Lawson AB Bansal S. Mind the scales: harnessing spatial big data for infectious disease surveillance and inference. J Infect Dis. (2016) 214:S409–s413. doi: 10.1093/infdis/jiw344, PMID: 28830109 PMC5144899

[105] Zhu L Huang L Fan L Huang J Huang F Chen J. Landslide Susceptibility Prediction Modeling Based on Remote Sensing and a Novel Deep Learning Algorithm of a Cascade-Parallel Recurrent Neural Network. Sensors. (2020) 20:1576. doi: 10.3390/s20061576 32178235 PMC7146231

[106] Khoury MJ Engelgau M Chambers DA Mensah GA. Beyond public health genomics: can big data and predictive analytics deliver precision public health? Public Health Genomics. (2018) 21:244–50. doi: 10.1159/000501465, PMID: 31315115 PMC6687519

[107] Wang Z Xia N Zhao X Gao X Zhuang S Li M. Evaluating urban vitality of street blocks based on multi-source geographic big data: a case study of Shenzhen. Int J Environ Res Public Health. (2023) 20:20. doi: 10.3390/ijerph20053821, PMID: 36900828 PMC10001719

[108] Segundo E Carrere-Molina J Aragon M Mallol-Parera R. Advancing geospatial preconception health research in primary care through medical informatics and artificial intelligence. Health Place. (2024) 89:103337. doi: 10.1016/j.healthplace.2024.103337, PMID: 39151214

[109] Krzyzanowski B Manson SM. Twenty years of the health insurance portability and accountability act Safe Harbor provision: unsolved challenges and ways forward. JMIR Med Inform. (2022) 10:e37756. doi: 10.2196/37756, PMID: 35921140 PMC9386597

[110] Wang J Kwan MP. Daily activity locations k-anonymity for the evaluation of disclosure risk of individual GPS datasets. Int J Health Geogr. (2020) 19:7. doi: 10.1186/s12942-020-00201-9, PMID: 32138736 PMC7059321

[111] Shen X Jiang H Chen Y Wang B Gao L. PLDP-FL: federated learning with personalized local differential privacy. Entropy (Basel). (2023) 25:25. doi: 10.3390/e25030485, PMID: 36981374 PMC10048376

[112] Guo S Yang J Long S Wang X Liu G. Federated learning with differential privacy via fast Fourier transform for tighter-efficient combining. Sci Rep. (2024) 14:26770. doi: 10.1038/s41598-024-77428-0, PMID: 39500977 PMC11538499

[113] Suwer S Ullah MS Probul N Maier A Baumbach J. Privacy-by-design with federated learning will drive future rare disease research. J Neuromuscul Dis. (2024) 6276:22143602241296276. doi: 10.1177/22143602241296276 PMC13141844 39973411

[114] Ajayakumar J Curtis A Curtis J. The utility of Zip4 codes in spatial epidemiological analysis. PLoS One. (2023) 18:e0285552. doi: 10.1371/journal.pone.0285552, PMID: 37256874 PMC10231782

[115] Ajayakumar J Curtis AJ Curtis J. Addressing the data guardian and geospatial scientist collaborator dilemma: how to share health records for spatial analysis while maintaining patient confidentiality. Int J Health Geogr. (2019) 18:30. doi: 10.1186/s12942-019-0194-8, PMID: 31864350 PMC6925902

[116] Araujo Navas AL Osei F Leonardo LR Soares Magalhaes RJ Stein A. Modeling Schistosoma japonicum infection under pure specification Bias: impact of environmental drivers of infection. Int J Environ Res Public Health. (2019) 16:176. doi: 10.3390/ijerph16020176, PMID: 30634518 PMC6351909

[117] Ponkilainen VT Uimonen M Raittio L Kuitunen I Eskelinen A Reito A. Multivariable models in orthopaedic research: a methodological review of covariate selection and causal relationships. Osteoarthr Cartil. (2021) 29:939–45. doi: 10.1016/j.joca.2021.03.020, PMID: 33933587

[118] Siegel K Dee LE. Foundations and future directions for causal inference in ecological research. Ecol Lett. (2025) 28:e70053. doi: 10.1111/ele.70053, PMID: 39831541

[119] Flaherty E Sturm T Farries E. The conspiracy of Covid-19 and 5G: spatial analysis fallacies in the age of data democratization. Soc Sci Med. (2022) 293:114546. doi: 10.1016/j.socscimed.2021.114546, PMID: 34954674 PMC8576388

[120] Kakampakou L Stokes J Hoehn A de Kamps M Lawniczak W Arnold KF. Simulating hierarchical data to assess the utility of ecological versus multilevel analyses in obtaining individual-level causal effects. BMC Med Res Methodol. (2025) 25:79. doi: 10.1186/s12874-025-02504-6, PMID: 40121398 PMC11929225

[121] Gao J May MR Rannala B Moore BR. Model misspecification misleads inference of the spatial dynamics of disease outbreaks. Proc Natl Acad Sci USA. (2023) 120:e2213913120. doi: 10.1073/pnas.2213913120, PMID: 36897983 PMC10089176

[122] Muralidharan V Ng MY AlSalamah S Pujari S Kalra K Singh R. Global initiative on AI for health (GI-AI4H): strategic priorities advancing governance across the United Nations. NPJ Digit Med. (2025) 8:219. doi: 10.1038/s41746-025-01618-x, PMID: 40269244 PMC12019307

[123] Kamarul Aryffin HA Bin Sahbudin MA Ali Pitchay S Abhalim AH Sahbudin I. Technological trends in epidemic intelligence for infectious disease surveillance: a systematic literature review. PeerJ Comput Sci. (2025) 11:e2874. doi: 10.7717/peerj-cs.2874, PMID: 40567698 PMC12192675

[124] Prakash Nayak P Pai BJ Govindan S. Leveraging geographic information system for dengue surveillance: a scoping review. Trop Med Health. (2025) 53:102. doi: 10.1186/s41182-025-00783-9 40760031 PMC12320306

[125] Zidane M Makky A Bruhns M Rochwarger A Babaei S Claassen M. A review on deep learning applications in highly multiplexed tissue imaging data analysis. Front Bioinform. (2023) 3:1159381. doi: 10.3389/fbinf.2023.1159381, PMID: 37564726 PMC10410935

[126] Wu Y Cheng Y Wang X Fan J Gao Q. Spatial omics: navigating to the golden era of cancer research. Clin Transl Med. (2022) 12:e696. doi: 10.1002/ctm2.696, PMID: 35040595 PMC8764875

[127] Maurya R Chug I Vudatha V Palma AM. Applications of spatial transcriptomics and artificial intelligence to develop integrated management of pancreatic cancer. Adv Cancer Res. (2024) 163:107–36. doi: 10.1016/bs.acr.2024.06.007, PMID: 39271261

[128] Al-Mansour FSH Almasoudi HH Albarrati A. Mapping molecular landscapes in triple-negative breast cancer: insights from spatial transcriptomics. Naunyn Schmiedeberg's Arch Pharmacol. (2025) 398:11125–43. doi: 10.1007/s00210-025-04057-3, PMID: 40119898

[129] Zuo C Zhu J Zou J Chen L. Unravelling tumour spatiotemporal heterogeneity using spatial multimodal data. Clin Transl Med. (2025) 15:e70331. doi: 10.1002/ctm2.70331, PMID: 40341789 PMC12059211

[130] Ayana G Dese K Daba Nemomssa H Habtamu B Mellado B Badu K. Decolonizing global AI governance: assessment of the state of decolonized AI governance in sub-Saharan Africa. R Soc Open Sci. (2024) 11:231994. doi: 10.1098/rsos.231994, PMID: 39113766 PMC11303018

[131] Qiu Y Hu Z. Data governance and open sharing in the fields of life sciences and medicine: a bibliometric analysis. Digit Health. (2025) 11:20552076251320302. doi: 10.1177/20552076251320302, PMID: 40013073 PMC11863247

[132] Abdel Magid HS Desjardins MR Hu Y. Opportunities and shortcomings of AI for spatial epidemiology and health disparities research on aging and the life course. Health Place. (2024) 89:103323. doi: 10.1016/j.healthplace.2024.103323, PMID: 39047648 PMC11402565

